# Survival Differences among Native-Born and Foreign-Born Older Adults in the United States

**DOI:** 10.1371/journal.pone.0037177

**Published:** 2012-05-16

**Authors:** Matthew E. Dupre, Danan Gu, James W. Vaupel

**Affiliations:** 1 Duke Clinical Research Institute, Duke University, Durham, North Carolina, United States of America; 2 Center for the Study of Aging and Human Development, Duke University, Durham, North Carolina, United States of America; 3 Population Division, United Nations, New York, New York, United States of America; 4 Max Planck Institute for Demographic Research, Rostock, Germany; University of Washington, United States of America

## Abstract

**Background:**

Studies show that the U.S. foreign-born population has lower mortality than the native-born population before age 65. Until recently, the lack of data prohibited reliable comparisons of U.S. mortality by nativity at older ages. This study provides reliable estimates of U.S. foreign-born and native-born mortality at ages 65 and older at the end of the 20^th^ century. Life expectancies of the U.S. foreign born are compared to other developed nations and the foreign-born contribution to total life expectancy (TLE) in the United States is assessed.

**Methods:**

Newly available data from Medicare Part B records linked with Social Security Administration files are used to estimate period life tables for nearly all U.S. adults aged 65 and older in 1995. Age-specific survival differences and life expectancies are examined in 1995 by sex, race, and place of birth.

**Results:**

Foreign-born men and women had lower mortality at almost every age from 65 to 100 compared to native-born men and women. Survival differences by nativity were substantially greater for blacks than whites. Foreign-born blacks had the longest life expectancy of all population groups (18.73 [95% confidence interval {CI}, 18.15–19.30] years at age 65 for men and 22.76 [95% CI, 22.28–23.23] years at age 65 for women). The foreign-born population increased TLE in the United States at older ages, and by international comparison, the U.S. foreign born were among the longest-lived persons in the world.

**Conclusion:**

Survival estimates based on reliable Medicare data confirm that foreign-born adults have longer life expectancy at older ages than native-born adults in the United States.

## Introduction

Life expectancy at birth in the United States ranks relatively low in international comparisons, behind countries such as Canada, Costa Rica, Japan, South Korea, and many European nations [1], [2]. However, life expectancy in the U.S. ranks appreciably higher at ages 65 and older [2], [3]. Perhaps fundamental to this paradox and our understanding of human longevity may be the impact of birthplace on adult mortality. In the last three decades of the 20^th^ century, the foreign-born population in the United States was one of the fastest growing segments of the population, exceeding 28 million (approximately 10% of the total U.S. population) by the year 2000 [4], [5]. During this period, researchers observed that the U.S. foreign-born population had better health and lower mortality relative to persons who were native born [6]–[12]. Although research on immigrant status and mortality has been limited at older ages, recent evidence from Singh and colleagues has shown that life expectancy at age 65 is approximately 1.5 years longer among the foreign-born population than among the U.S. born population [13]. There also is evidence to suggest that differences were greater among men than women and that the survival disparities widened from 1980 to 2000 [13].

Until recently, the lack of data prohibited reliable comparisons of U.S. mortality by nativity at older ages. Current estimates of life expectancy at older ages combine vital statistics and census enumerations to approximate death counts (numerators) and population size (denominators), respectively [14]. As a result, the accuracy of such rates are unknown and questions remain about how the immigrant population impacts mortality heterogeneity and survival expectations in later life. The purpose of this study was to provide accurate population estimates of survival for foreign-born and native-born adults aged 65 and older at the end of the last century.

## Methods

We used newly available data from Medicare records linked with Social Security Administration (SSA) files to examine mortality differences in nativity by sex, race, and age. Official estimates of U.S. mortality at older ages are based on data of historically poor quality [14], which approximate death counts and population size from two sources (i.e., vital statistics and U.S. census data). Research has established that the use of Medicare data to estimate old-age mortality is more accurate than traditional data derived from multiple sources [15], [16]. Enrollment in Medicare is nearly universal at older ages and covers more than 95% of the U.S. population aged 65 and older, including the institutionalized [17]. The scientific consensus is that data from enrollees in Medicare Part B (medical insurance) provide the most accurate age reporting and population counts because the program requires monthly payments for services [16], [17]. The cancellation or non-receipt of premium payments results in the termination of enrollment in the program, which unlike Medicare Part A (hospital insurance), provides an important mechanism to exclude decedents from the population. Further details of the Medicare data and their quality, particularly for estimating mortality, have been reported extensively elsewhere [15]–[17].

The current calculations were based on computerized Medicare Part B data that were merged with Social Security Administration (SSA) records for more than 30 million U.S. elderly in 1995. Although we acknowledge the age of the data, the current study uses the only population-wide cohort data currently available to reliably estimate U.S. mortality differences by nativity, sex, and race at older ages. Prior population-based studies of mortality encounter comparable lags in the availability of suitable data for analysis [10], [13], [18]–[21]. Unlike existing data, the analyses in this study are based on the only Medicare Part B data that have been linked with the SSA Numident file for all enrollees to provide detailed information on place of birth. These unique data are the result of enormous coordination and effort by the SSA, Health Care Financing Administration (now the Centers for Medicare and Medicaid Services), National Institutes on Aging, and Duke University. Medicare data linked with SSA records in more recent years are currently not available for analysis.

Deaths were recorded in the SSA Numident file and linked to Medicare data using social security numbers (SSN) [16], [17]. Sex and race were provided by SSN applicants and were included in the analyses to account for known demographic variations in mortality at older ages [22]. Place of birth was ascertained from SSN applications and was provided in the Numident file. Although nativity was determined for the majority of SSN applicants, there were cases that could not be determined due to erroneous or missing information. Consistent with previous research using SSA-linked Medicare records [23], [24], approximately 15% of men and 23% of women had missing information on birthplace. On average, enrollees with missing data were more likely to be older and white compared to persons with identifiable nativity. Improvements in vital registration and data recording were also reflected in rates of missing that diminished over time. Missing patterns corresponded closely with the computerization of the Numident file that occurred during the mid-1970s and mainly affected persons who turned 65 before 1977 [17], [23], [24]. We also know from census enumerations and the distributional makeup of enrollees that the vast majority of persons with unknown nativity in our study were born in the United States [4], [23], [25]. We excluded persons over 100 years of age because the accuracy of U.S. death rates at these ages has been challenged [3], [14].

Preliminary analyses of the Medicare data were performed. First, we calculated age-specific survival probabilities for all Medicare enrollees by sex and race and compared them to official estimates reported by the National Center for Health Statistics (NCHS) [26]. Results confirmed that survival probabilities in the NCHS data corresponded closely to estimates from the Medicare data when persons with unknown birthplace were included among the native-born population (Figure S1). Second, we estimated survival probabilities for men and women by race with missing nativity data and compared them to the estimates from the Medicare and NCHS data in 1995 (Figure S1). Overall, cumulative survival rates among women with unknown nativity were nearly identical with estimates from the Medicare and NCHS sources; for men, cumulative survival rates among those with missing data were lower (approximately 30% lower at some ages) compared to the other sources. Although deviations were apparent among white and black men, the overall rates of those with unknown place of birth were low (≤15%). A third set of analyses examined potential age-misreporting in the foreign-born Medicare data. We compared life expectancies between foreign-born populations from countries with excellent vital record data (Sweden, France, Japan, UK, Spain, Germany, Hungary, and Denmark) and from the remaining countries with data that are known to be less reliable [27]. As previously demonstrated, life expectancies in countries with poorer data were slightly overestimated for the foreign born (Figure S2) [27]. The discrepancies were similar for men and women and were relatively small. Based on these tests, we are confident that the current estimates are not severely biased by missing data or age misreporting.

Period life tables were used to examine age-specific death rates and five-year survival probabilities among Medicare enrollees by nativity, sex, and race in 1995 [28]. We also estimated life expectancies at select ages by nativity group and compared the U.S. foreign-born population to other nations to assess the magnitude of the survival advantage [1] at age 65. The contribution of the foreign-born population to U.S. total life expectancy (TLE) at older ages was assessed. Details of the life table methods and calculations of 95% confidence intervals (CI) are provided online (Appendix S1). All analyses were conducted using Stata version 11 (StataCorp, College Station, Texas) and Excel version 2007 (Microsoft Corporation, Redmond, Washington).

### Ethics Statement

The institutional review board at Duke University reviewed and approved the data used in this study.

## Results

**Figure 1:**
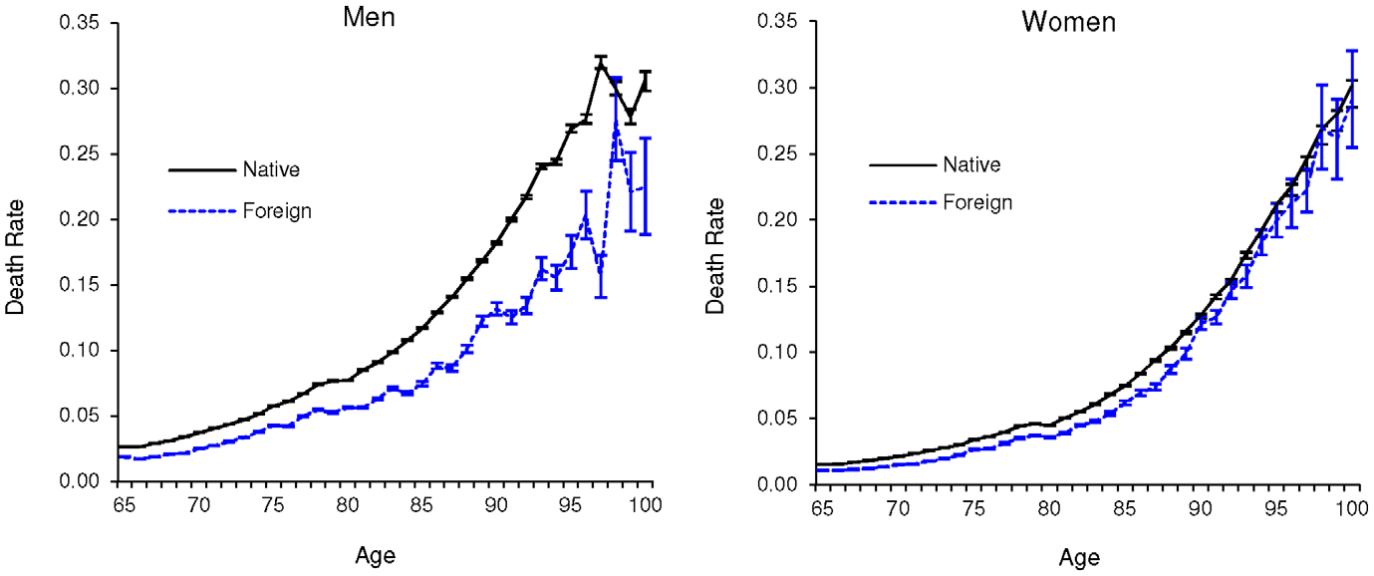
Age-Specific Death Rates and 95% Confidence Intervals for Native-born and Foreign-born Populations in the United States in 1995.

Table 1 provides the sample sizes and total number of deaths at selected ages for the native-born and foreign-born populations in the SSA-linked Medicare data. Figure 1 shows that the age-specific death rates for the older foreign-born population were lower than the native-born population at most ages in 1995. These results confirm the foreign-born survival advantage at older ages and suggest that the mortality gap was greatest among men. There was also evidence that differences in mortality widened between nativity groups across age, particularly among men.

**Table 1:** Sample Size and Number of Deaths at Selected Ages for Native-born and Foreign-born Populations in the United States in 1995.

		Native Born		Foreign Born	
		White	Black	White	Black
Men		Sample Size			
At ages	65	3,628,085	328,913	297,481	17,061
	70	3,112,616	260,210	266,509	12,053
	75	2,147,196	163,895	150,921	6,871
	80	1,292,127	98,835	79,194	3,016
	85	821,057	67,267	25,255	1,140
Women					
At ages	65	4,246,709	449,369	413,738	24,452
	70	4,065,131	388,883	368,627	18,108
	75	3,240,219	285,615	216,077	11,908
	80	2,393,159	206,922	131,036	6,736
	85	2,212,959	190,729	107,063	3,747
Men		Number of Deaths			
At ages	65	96,546	14,143	5,606	377
	70	127,020	15,362	8,154	393
	75	136,070	13,795	7,648	335
	80	112,632	9,526	5,261	226
	85	134,597	10,556	2,901	106
Women					
At ages	65	65,950	10,886	4,710	339
	70	97,523	13,723	6,697	301
	75	124,162	14,007	7,059	382
	80	130,422	12,373	6,046	239
	85	274,497	21,457	12,871	310

The upper panel in Table 2 shows the five-year survival probabilities at ages 65 to 85 by sex and race in 1995. At age 65, 91.07 (95% CI, 90.85, 91.30) percent of foreign-born white men and 94.47 (95% CI, 94.32, 94.63) percent of foreign-born white women survived to age 70, compared to only 87.49 (95% CI, 87.42, 87.56) and 92.49 (95% CI, 92.43, 92.54) percent of native-born white men and women, respectively. The discrepancies in five-year survival were larger among blacks and at ages 70 to 85. Differences in survival probabilities between nativity groups were generally greater among men than among women, and at every age, foreign-born women had the highest five-year survival rates. The native-born black population had the lowest five-year survival rates at age 65 (80.54 [95% CI, 80.25, 80.82] for men and 88.52 [95% CI, 88.32, 88.73] for women), although by age 85, they had survival probabilities that exceeded their native-born white counterparts (50.23 [95% CI, 49.36, 51.10] for men and 63.68 [95% CI, 63.14, 64.23] for women). Nativity differences in five-year survival increased across age in both absolute and relative levels (percent differences), particularly among blacks.

**Table 2:** Five-Year Survival Probabilities and Life Expectancies at Selected Ages for Native-born and Foreign-born Populations in the United States in 1995.

		Native Born				Foreign Born			
		White		Black		White		Black	
Men		Five-year survival in percentages (95% CI)							
At ages	65	87.49	(87.42–87.56)	80.54	(80.25–80.82)	91.07	(90.85–91.30)	89.97	(89.01–90.93)
	70	81.45	(81.36–81.54)	74.33	(73.98–74.68)	85.73	(85.44–86.01)	84.85	(83.47–86.23)
	75	72.86	(72.73–72.98)	65.70	(65.24–66.17)	78.21	(77.77–78.64)	78.91	(76.91–80.92)
	80	64.53	(64.37–64.70)	61.47	(60.86–62.08)	73.18	(72.56–73.80)	70.61	(67.39–73.83)
	85	49.61	(49.37–49.85)	50.23	(49.36–51.10)	63.10	(61.69–64.51)	69.09	(63.11–75.06)
Women									
At ages	65	92.49	(92.43–92.54)	88.52	(88.32–88.73)	94.47	(94.32–94.63)	93.46	(92.79–94.13)
	70	88.64	(88.57–88.71)	83.80	(83.55–84.05)	91.33	(91.13–91.53)	92.04	(91.17–92.90)
	75	82.56	(82.48–82.65)	78.31	(77.99–78.63)	85.35	(85.03–85.67)	85.37	(84.02–86.73)
	80	76.04	(75.92–76.15)	74.03	(73.64–74.43)	79.78	(79.32–80.23)	84.27	(82.43–86.10)
	85	62.29	(62.13–62.45)	63.68	(63.14–64.23)	66.77	(66.01–67.53)	74.85	(71.36–78.35)
Men		Life expectancy in years (95% CI)							
At ages	65	15.86	(15.84–15.88)	13.58	(13.51–13.64)	18.64	(18.52–18.75)	18.73	(18.15–19.30)
	70	12.75	(12.73–12.77)	11.25	(11.18–11.31)	15.21	(15.09–15.33)	15.55	(14.94–16.15)
	75	10.07	(10.05–10.09)	9.27	(9.20–9.33)	12.31	(12.18–12.43)	12.83	(12.16–13.51)
	80	7.88	(7.86–7.90)	7.84	(7.76–7.91)	10.03	(9.88–10.17)	10.58	(9.80–11.37)
	85	5.83	(5.81–5.85)	6.22	(6.14–6.31)	7.79	(7.61–7.94)	8.99	(8.01–9.97)
Women									
At ages	65	19.73	(19.71–19.75)	18.11	(18.04–18.18)	21.36	(21.28–21.44)	22.76	(22.28–23.23)
	70	16.12	(16.10–16.14)	15.12	(15.06–15.19)	17.45	(17.37–17.53)	19.17	(18.68–19.65)
	75	12.85	(12.83–12.87)	12.55	(12.49–12.62)	13.86	(13.78–13.93)	15.59	(15.09–16.08)
	80	10.01	(9.99–10.03)	10.34	(10.27–10.40)	10.78	(10.70–10.86)	12.81	(12.28–13.34)
	85	7.35	(7.33–7.36)	8.07	(8.00–8.14)	7.85	(7.77–7.93)	9.72	(9.15–10.29)

Abbreviation: CI, confidence interval.

In terms of life expectancy (Table 2, lower panel), foreign-born white men (18.64 [95% CI, 18.52, 18.75]) lived approximately three years longer at age 65 than native-born white men (15.86 [95% CI, 15.84, 15.88]) and foreign-born white women (21.36 [95% CI, 21.28, 21.44]) lived approximately two years longer at age 65 than native-born white women (19.73 [95% CI, 19.71, 19.75]). Foreign-born black men and women had the longest life expectancy and were estimated to live an additional 18.73 (95% CI, 18.15, 19.30) and 22.76 (95% CI, 22.28, 23.23) years after age 65. Foreign-born black men (8.99 [95% CI, 8.01, 9.97]) lived almost twice as long at age 85 than native-born white men (5.83 [95% CI, 5.81, 5.85]). Results also suggest that foreign-born black women lived longer at age 80 (12.81 [95% CI, 12.28, 13.34] years) than native-born black men who were 10 years younger (11.25 [95% CI, 11.18, 11.31] years).

**Figure 2:**
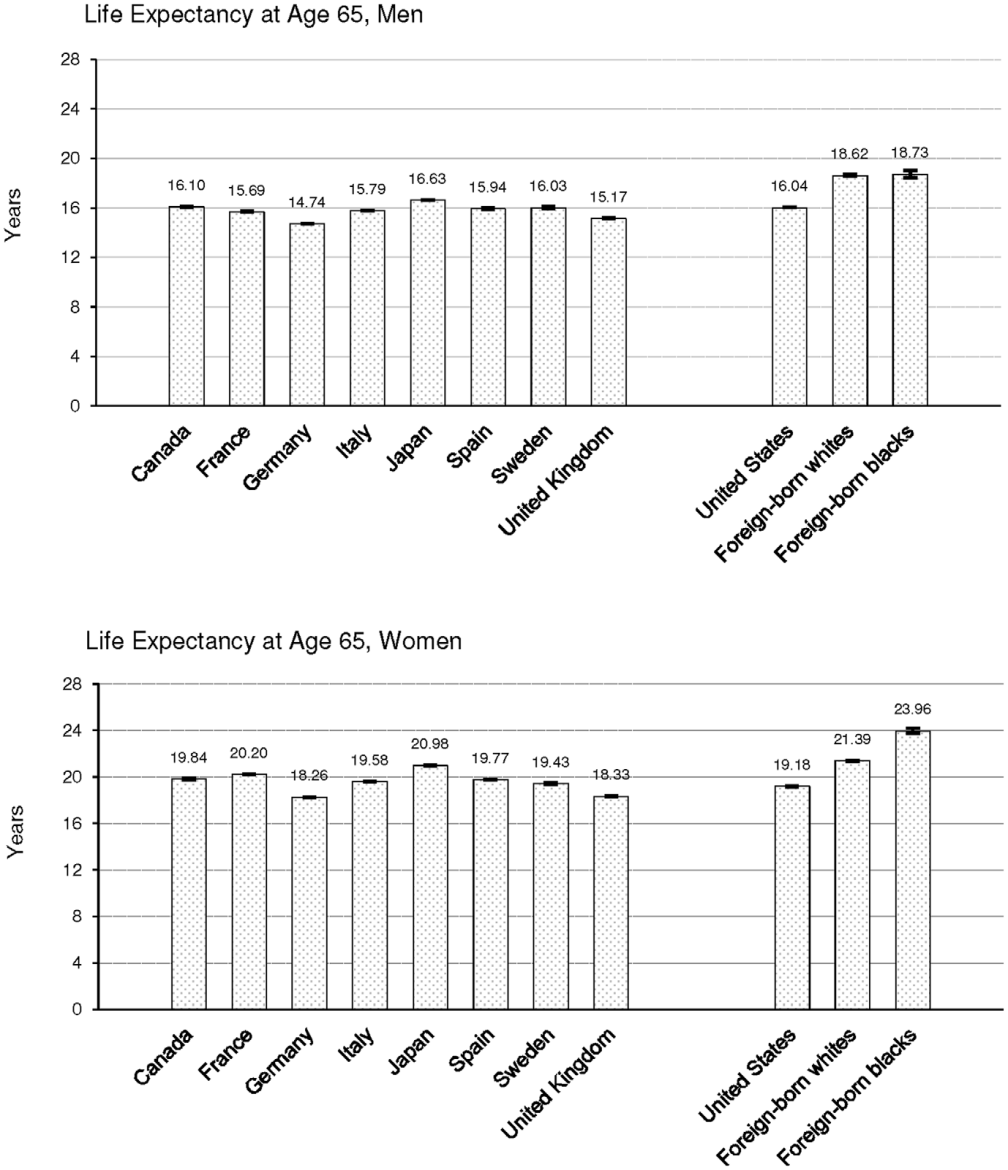
Life Expectancies and 95% Confidence Intervals at Age 65 for Selected Countries and for the U.S. Total and Foreign-born Populations in 1995.

By international comparison (Figure 2), the overall life expectancies of foreign-born men and women at age 65 in the United States surpassed the overall life expectancies of Canada, France, Germany, Italy, Japan, Spain, Sweden, and the United Kingdom at age 65. In fact, the Medicare estimates suggest that U.S. foreign-born black men and women had life expectancies that were approximately two years longer than men (18.73 [95% CI, 18.44, 19.30] vs. 16.63 [95% CI, 16.60, 16.66]) and women (22.76 [95% CI, 22.28, 23.23] vs. 20.98 [95% CI, 20.96, 21.00]) from Japan, respectively.

**Figure 3:**
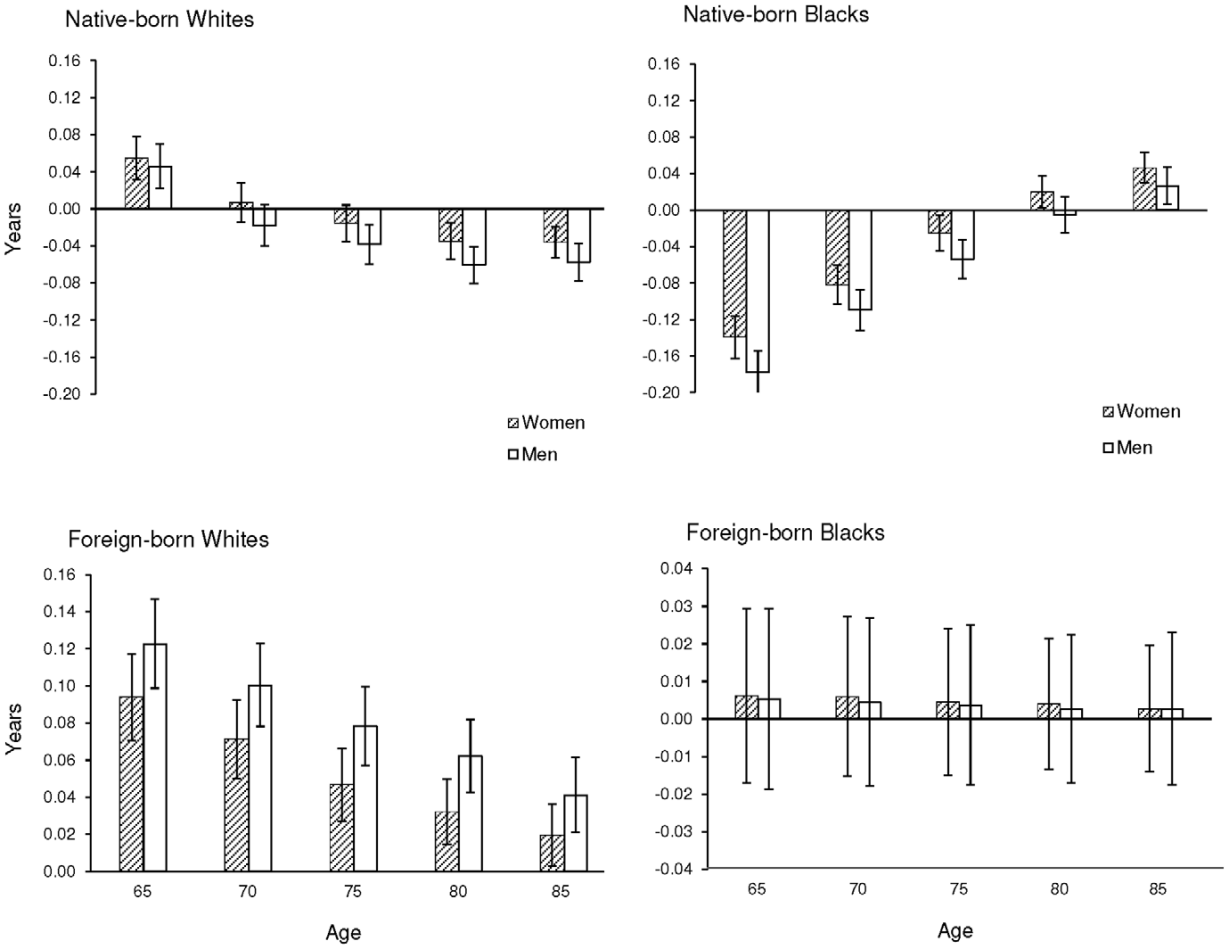
Years Added to U.S. Total Life Expectancy and 95% Confidence Intervals at Selected Ages for Native-born and Foreign-born Populations in 1995.

Although the foreign born represented only a small percent of U.S. older adults in the last decades of the 20^th^ century, their contribution to total life expectancy (TLE) at ages 65 and older was appreciable. Figure 3 shows that the foreign-born population increased TLE at all ages for whites (but not blacks) and that the contributions were similar for men and women. At age 65, the high life expectancies of foreign-born white men added 0.12 (95% CI, 0.10, 0.14) years to TLE for men and foreign-born white women added 0.09 (95% CI, 0.07, 0.11) years to TLE for women. Patterns were similar for foreign-born blacks, although the contribution levels were less due to their smaller population sizes. Overall, the foreign-born contributions were substantial in 1995 considering that life expectancy at age 65 improved by only 1.5 years for men and 0.6 years for women from 1980 to 1995 [29].

## Discussion

Newly available data from SSA-linked Medicare records provided a unique opportunity to reliably compare survival rates between U.S. foreign-born and native-born elderly populations at the end of the last century. Results from period life tables demonstrated a clear foreign-born survival advantage among older adults in the United States in 1995. Survival differences between foreign-born and native-born populations were large and consistent at ages 65 and older, particularly among men and blacks. Overall, we found that the foreign-born elderly population contributed to U.S. life expectancy and were among the longest-lived in the world.

Foreign-born blacks had the longest life expectancy at older ages compared to the other population groups that were studied. To our knowledge, this is the most reliable documentation of what appears to be exceptional longevity among older U.S. blacks born outside of this country. This finding is in contrast to the well-documented disadvantages and correlated health risks among native-born blacks. It is possible that the black foreign-born mortality advantage may amplify what has been described as the black-white mortality crossover that is often observed at old ages. Unlike previous research, the current estimates were based on Medicare Part B data that have more accurate age reporting than most studies showing lower mortality among older blacks compared to older whites. If poor data quality among blacks were indeed in question, native-born blacks also would exhibit similar (or superior) survival compared to whites—but there is little evidence of this. Nevertheless, we cannot rule out data quality or the influence of other unobserved factors that are unique to foreign-born blacks.

A somewhat unexpected finding was the degree to which the white foreign-born population increased U.S. life expectancy. The white foreign-born contributions to TLE in 1995 were sizeable relative to the population sizes and life expectancies of each sex and age group. Although the U.S. foreign-born population was one of the fastest growing segments of the population during the last decades of the 20^th^ century [4], [5]; it is unknown whether their contribution to U.S. life expectancy will persist in the early 21^st^ century. It is also unknown how the relatively young foreign-born Hispanic population will shape U.S. mortality in the future. Researchers should consider these demographic factors and the role of unobserved heterogeneity in socio-behavioral risks across nativity groups that may explain the survival differentials [30], [31].

The observation that the U.S. foreign-born were among the longest-lived older adults in the world was remarkable but not unexpected. According to the United Nations, the number of immigrants in the United States is more than triple any other nation in the world—a feature that has come to define the diversity of America in the 20^th^ century [32]. Historically, the U.S. foreign-born population was comprised of predominantly healthy, young (working-age), European migrants who resided and worked in rapidly modernizing urban areas. The finding that foreign-born white men contributed to U.S. life expectancy corroborates the possibility of health selection (i.e., healthy men migrating for occupational reasons). It also is plausible that survival is greater at older ages for those born in a foreign country than for those born in the United States because of a biological or acquired resilience (i.e., physiological/psychological robustness) that promotes relatively low mortality at advanced ages. However, what is unique is that the low mortality of the foreign born at older ages did not follow a period of elevated risks at younger ages that typically characterizes relative mortality advantages in late life [13], [33]. To be sure, we await validation of these findings before speculating further about their underlying causes.

A key strength of this study is that the Medicare data allowed us to estimate survival rates from a single source and are more accurate than current estimates based on vital statistics and census enumerations. Unfortunately, the data are limited as an empirical resource to elucidate the factors that may explain the findings. Central to this shortcoming is whether and to what degree the low mortality of foreign-born older adults is an artifact of selective return-migration among foreign-born adults with advanced illness and anticipated death. The so-called “salmon bias” has been proposed to explain the mortality advantage of U.S. Hispanics, namely Mexican Americans [34]; however, its impact on mortality at advanced ages and among other racial and ethnic immigrants is not well understood. Contrary to perception, the majority of older foreign-born adults at the end of the last century were from Europe (39%) and Asia (22%) and not from geographically proximate nations such as Mexico, Cuba, or Canada [25]. Moreover, almost two-thirds of the older foreign-born lived in the United States for more than 30 years and are much less likely to leave the country in late life than the younger foreign-born [35], [36]. We also know that foreign-born and native-born populations were not covered equally by health insurance at ages 65 and older. In 2000, approximately 96% of the native-born population was enrolled in Medicare, compared to 90% of the foreign-born population [25]. Therefore, foreign-born adults who were most likely to migrate out of the United States at older ages (the uninsured) were not included in our study of Medicare enrollees. In other words, uninsured foreign-born adults were omitted from numerators *and* denominators when estimating mortality rates for the foreign-born populations. Considering these factors, the potential influence of migration bias on the results is presumably minimal. Indeed, a recent analysis of SSA data showed that a salmon bias was too negligible to impact the Hispanic mortality advantage in the United States [21]. Nevertheless, we recognize that further consideration of migration is critical to understanding how nativity impacts adult mortality and merits further research.

We also recognize that the period life table estimates are from 1995. However, it is important to underscore the relevance of these findings in light of current science and to current public health surveillance. First, this study provides the most accurate and current population estimates of survival for foreign-born and native-born adults aged 65 and older. Until now, the lack of data has prohibited reliable comparisons of U.S. mortality by nativity at older ages. Second, estimates of life expectancy at older ages in the United States have changed little over the past several decades—i.e., by only 0.4–1.3 years at age 65 from 1995 to 2005 [26], [37], regardless of sex and race. In fact, the most recent official estimates of U.S. life expectancy at age 65 are only as current as 2005. Third, the findings are unique because they are based on nearly the entire U.S. population (≥95%) ages 65 and older, and to the best of our knowledge, these data are the only source of detailed information on place of birth for such a large number of U.S. adults (n>30,000,000). In sum, this study provided an unprecedented opportunity to (a) accurately determine nativity for almost all U.S. older adults and (b) estimate mortality rates from a single source—which are more accurate than existing estimates derived from multiple sources (including the official U.S. estimates by NCHS in 2005).

Age misreporting is an unavoidable source of bias in mortality estimates at older ages [38], [39] and the current data are not immune. The accuracy and availability of documentation provided to enter the United States (e.g., birth certificates) varied significantly by country and region. However, preliminary analyses of foreign-born populations from countries with excellent vital record data and less reliable data suggested that the major findings were not simply an artifact of age misreporting. Another potential source of bias was missing data on place of birth. Supplemental analyses showed that missing data were relatively low and did not severely bias the observed survival patterns. However, we acknowledge that missing and erroneous data are important to consider when interpreting the magnitude of nativity differences in mortality, and until new and improved Medicare data with SSA linkages to nativity become available, we remain cautious in our conclusions.

The results of this analysis have potential relevance for health policy. Approximately 99% of native-born adults aged 65 and older had health insurance at the end of the last century (primarily through Medicare); compared to 94% of foreign-born elders [25]. However, the nativity gap in health care coverage at all ages is far more pronounced. During the same period, 87% of the native-born population had access to health insurance; compared to less than 67% of the foreign-born population [40]. The fact that foreign-born adults seem to be living longer than native-born adults despite having lower rates of health insurance suggests that factors other than access to health care are important to the longevity of older U.S. immigrants [3], [41]. We encourage researchers to make additional linkages to data (e.g., cause-specific mortality) to enrich our understanding of these findings and their implications for public health and medical care. In the current era of U.S. health care reform, it is important to reassess access and the delivery of quality care to all segments of the population and its ever-expanding landscape of diversity.

Fundamental to questions about human aging, from fetal origins to late life, is whether and how birthplace modulates disparities in healthy longevity [42]–[47]. Using unique data from SSA-linked Medicare records, we provided the first reliable documentation of differences in age-specific survival and life expectancy by nativity, sex, and race at a significant period. Our estimates validated the foreign-born survival advantage and demonstrated that the older immigrant population improved U.S. life expectancy at the end of the last century. It is not yet known how younger foreign-born cohorts will impact future mortality heterogeneity and survival expectations in the United States.

## Supporting Information

Figure S1 **Cumulative Survival Probabilities in 1995 for All Medicare Enrollees, for Persons with Missing Data on Nativity, and for the U.S. Population (1).**
(TIF)

Figure S2 **Life Expectancies and 95% Confidence Intervals at Age 65 among Foreign-born Populations from Countries with Excellent Data and Less Reliable Data in 1995.**
(TIF)

Appendix S1 **Period Life Table Calculations.**
(PDF)
