# Supplementary figures and images for: Survival Differences among Native-Born and Foreign-Born Older Adults in the United States
Source: PLoS One. 2012 May 16;7(5):e37177. doi: 10.1371/journal.pone.0037177 (PMC3353911; doi:10.1371/journal.pone.0037177)

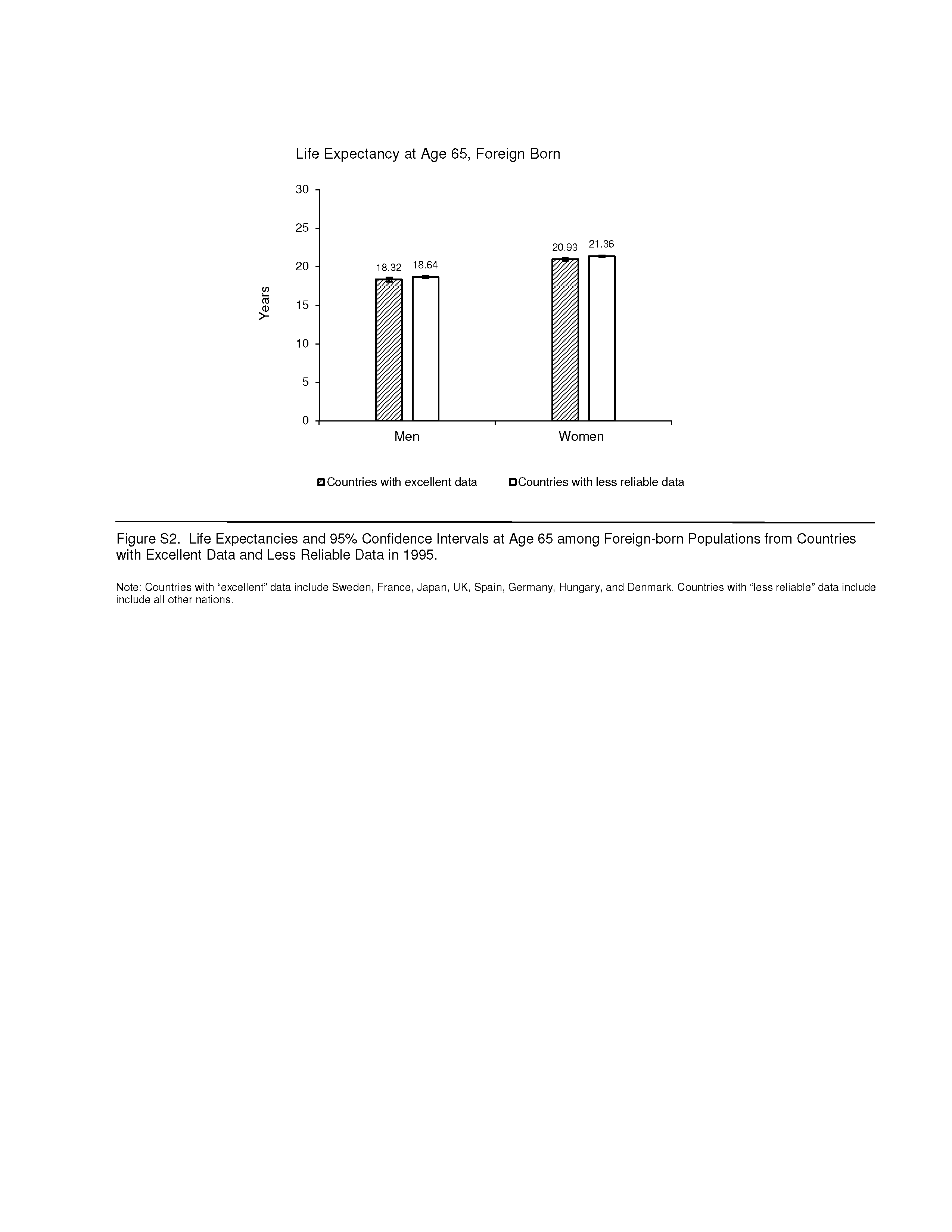

Supplement: Figure S2 — Life Expectancies and 95% Confidence Intervals at Age 65 among Foreign-born Populations from Countries with Excellent Data and Less Reliable Data in 1995. (TIF) [file pone.0037177.s002.tif]
