# Supplementary material for: Survival Differences among Native-Born and Foreign-Born Older Adults in the United States
Source: PLoS One. 2012 May 16;7(5):e37177. doi: 10.1371/journal.pone.0037177 (PMC3353911; doi:10.1371/journal.pone.0037177)
Supplement: Appendix S1 — Period Life Table Calculations. (PDF) [file pone.0037177.s003.pdf]

Appendix S1. Period Life Table Calculations

Death rates were calculated as: $m_x = D_x / P_x$, where m_x represents the death rate at age x in 1995; D_x is the number of deaths at age x in 1995; P_x (=[0.5*($P(x$, 1994)+$P(x$,1995))] is the average size of the population at age x in 1995; $P(x$, 1994) is the population at age x at the end of 1994 or at the beginning of 1995; and $P(x$, 1995) is the population at age x at the end of 1995.

The standard error (SE) for the death rate m_x was calculated using the formula:[2(p79)]

SE(m_x)=$\sqrt{\frac{1}{P_x}(m_x(1-q_x)}$ and the 95% confidence interval (CI) for the death rate at age x is m_x ± 1.96SE(m_x), where q_x is the probability of death at age x in 1995.

Five-year survival probabilities by age, nativity, sex, and race were calculated as: ${}_5p_x$=1-${}_5q_x$, where ${}_5p_x$ represents the five-year survival probability from age x to age (x+5), and ${}_5q_x$ is the probability of death from age x to age (x+5).

The SE of the five-year survival probability was estimated using the formula:[2(p154)]

SE(${}_5p_x$)=$\sqrt{\frac{{}_5q_x^2(1-{}_5q_x)}{{}_5D_x}}$ and the 95% CI is ${}_5p_x$ ± 1.96SE(${}_5p_x$), where ${}_5D_x$ is the number of deaths in the age interval [x,x+5).

To estimate cumulative survival probabilities in the Medicare and National Center for Health Statistics (NCHS) data we used the formula: ${}_xp_{65} = \frac{l_x}{l_{65}}$, where l_x is the number of survivors reaching age x.

The SE of the cumulative survival probabilities in the Medicare and NCHS data was estimated as: [1,2(157)]

SE(${}_xp_{65}$)=$\sqrt{{}_xp_{65}^2 \sum_{i=x}^{x-n} {}_np_i^{-2} S_{{}_np_i}^2}$, where ${}_xp_{65}$ is the cumulative survival from age 65 to age x, and $S_{{}_np_i}^2$ (=SE^2(${}_np_i$)) is the variance of the survival probability from age i to age $(i+n)$, and n is the age interval (for Medicare data we used n=1; for NCHS data we used n=5 because the number of deaths is not available in single years).

We used $e_x = \frac{T_x}{l_x} = \frac{\sum_{i=x}^{\omega-1} {}_5L_i}{l_x}$ (x=65, 70, 75, 80, 85) to estimate average life expectancy, where l_x is the number of persons surviving to age x and ${}_5L_x$ is the number of person-years lived in the age interval [x,x+5) by l_x, T_x is the total person-years lived after age x *by* l_x.

We used the formula:[2(163)] SE(e_x)=$\sqrt{\sum_{i=x}^{\omega-1}[{}_ip_x^2(2.5+e_{i+5})^2 \frac{{}_5q_i^2(1-{}_5q_i)}{{}_5D_i}]}$ (i, x=65, 70, 75, 80, 85) to estimate the standard error for life expectancy at age x, where ${}_ip_x$ is the survival probability from age x to age i, e_x is life expectancy at age x and $\omega-1$ is the highest age group.

To estimate cumulative survival probabilities in the Medicare and National Center for Health Statistics (NCHS) data we used the formula: [1,2(157)] SE(${}_x p_{65}$)= $\sqrt{{}_x p_{65}^2 \sum_{i=x}^{x-n} {}_n p_i^{-2} S_{{}_n p_i}^2}$, where ${}_x p_{65}$ is the cumulative survival from age 65 to age x, n is the age interval (for Medicare data we used n=1; for NCHS data we used n=5 because the number of deaths is not available in single years), and $S_{{}_n p_i}^2$ (=SE^2(${}_n p_x$)) is the variance of the survival probability from age x to age (x+n).

For the contribution to total life expectancy (TLE) for a given subpopulation (e.g., foreign-born whites), we used the difference ($\Delta_{(e_x - e'_x)} = e_x - e'_x$) between the actual life expectancy of the total U.S. population at age x (e_x) and the expected life expectancy of the total U.S. population at age x(e'_x) if the given subpopulation experienced the age-specific mortality rates of the actual total U.S. population. In other words, for a given subpopulation, if it has lower mortality rates than the total actual U.S. population, e'_x will be smaller than e_x and the difference ($\Delta_{(e_x - e'_x)}$) will be positive, indicating that the subpopulation makes a positive contribution to the TLE of the U.S. population, and so on.

The 95% CIs of the contributions to TLE were estimated by: $\Delta_{(e_x - e'_x)}$ ± 1.96SE($\Delta_{(e_x - e'_x)}$), where SE($\Delta_{(e_x - e'_x)}$) is the square root of the summation of the two variances of life expectancies e_x and e'_x , that is, $\sqrt{SE^2(e_x) + SE^2(e'_x)}$.[2(p164)]

References

1. National Center for Health Statistics (1998) Vital statistics of the United States, 1995, preprint of vol II, mortality, part A sec 6 life tables. Hyattsville, Maryland.
2. Chiang CL (1984) The Life Table and its Applications, Malabar, FL: Robert E. Krieger Publishers.
